# Efficacy of Statin Therapy Related to Baseline Renal Function in Patients with Rheumatic Heart Disease Undergoing Cardiac Surgery

**DOI:** 10.1155/2018/5972064

**Published:** 2018-04-15

**Authors:** Rongjun Zou, Wanting Shi, Jun Tao, Xifeng Lin, Dingwen Zhang, Songran Yang, Ping Hua

**Affiliations:** ^1^Department of Cardio-Vascular Surgery, Sun Yat-sen Memorial Hospital, Sun Yat-sen University, Guangzhou 510120, China; ^2^Department of Gastroenterology, Fifth Affiliated Hospital, Sun Yat-sen University, Zhuhai 519000, China; ^3^The Biobank of Sun Yat-sen Memorial Hospital, Sun Yat-sen University, Guangzhou 510120, China; ^4^Guangdong Province Key Laboratory of Brain Function and Disease, Zhongshan School of Medicine, Sun Yat-sen University, Guangzhou 510080, China

## Abstract

**Background:**

Renal impairment increases the risk of cardiovascular events and perioperative complications in patients with heart valve disease. This study aimed to determine the perioperative benefit of statin treatment related to baseline renal function in patients with rheumatic heart disease (RHD) who had cardiac surgery.

**Methods and Results:**

We performed a retrospective study on 136 patients with RHD who underwent valve replacement surgery. The mean age of the patients was 56.2 years, 59.6% were female, 8.8% patients had diabetes mellitus, and 27.2% of patients had hypertension. Overall, 3 patients died, 2 underwent reoperation, and 25 underwent thoracentesis during the study period. For patients with renal impairment, there was a higher risk of thoracic puncture (odds ratio [OR]: 3.33; 95% confidence interval [CI]: 1.36, 8.11; *P* < 0.01) and a longer time of drainage (difference in means: 1; 95% CI: 0.88, 1.12; *P* < 0.01), intensive care unit (ICU) stay (difference in means: 0.2; 95% CI: 0.17, 0.23; *P* = 0.02), and hospital stay (difference in means: 6.6; 95% CI: 6.15, 7.05; *P* < 0.01) compared with normal renal function. Furthermore, statins were associated with a reduction in drainage time (difference in means: −1.50; 95% CI: −1.86, −1.14; *P* = 0.02), ICU stay (difference in means: −0.30; 95% CI: −0.40, −0.20; *P* = 0.05), and hospital stay (difference in means: −5.40; 95% CI: −6.57, −4.23; *P* < 0.01) in patients with renal impairment (interaction, *P* ≤ 0.05 for all), but not in those with normal renal function.

**Conclusion:**

Statins have a greater clinical benefit in perioperative cardiac surgery with renal impairment. Statins are associated with a comparatively lower risk of thoracic puncture, as well as a reduced trend toward a reduction in drainage time, ICU stay, and hospital stay.

## 1. Introduction

Rheumatic heart valve disease (RHD) is a chronic disease and a major challenge, with more than 345,000 deaths per year worldwide [1]. The number of patients with RHD is continuing to increase, especially in developing countries. Additionally, RHD is prone to arrhythmia, heart failure, and pathologic structural changes and is not conducive to clinical management at postoperation. Chronic kidney disease (CKD) is a major risk factor for cardiovascular disease because of fluid overload, electrolyte abnormalities, and metabolic acidosis causes. CKD is independently associated with perioperative bleeding and the safety of cardiac surgery [2]. Furthermore, approximately 30% of patients were reported to have acute renal dysfunction after cardiac surgery, especially in those with CKD before surgery [3, 4].

Recent studies have shown that the early stages of CKD are significantly associated with local microinflammation and proosteogenic molecules of vascular calcification [5]. Statins, which are compounds that inhibit 3-hydroxy-3-methyl-glutaryl-CoA (HMG-CoA) reductases, are expected to reduce the risk of acute renal injury following cardiac surgery. Statins play an important role in antioxidant and anti-inflammatory effects [6, 7]. Various benefits associated with statins and cardiac surgery have been reported based on risk factors that were identified in clinical studies and cohort studies [8, 9]. However, the clinical efficacy of thoracic drainage time, associated with baseline renal function, has not been extensively investigated in patients with RHD.

Therefore, the present study aimed to determine the perioperative benefit of statin treatment in patients with cardiac surgery and its association with baseline renal function. We recorded the cardiopulmonary bypass (CPB) time, aortic cross-clamp time, drainage time, intensive care unit (ICU) stay, hospital stay, and the rates of perioperative reoperation, thoracentesis, and mortality. We conducted a retrospective analysis of patients with RHD, who are more likely to have abnormal hemodynamics and who had cardiac surgery in relation to baseline renal function. We compared the effects of statins with nonstatins therapy using data obtained from the cohort study in the perioperative period.

## 2. Methods

### 2.1. Study Design

The present study was a retrospective, single-center study. Patients in the statin group were given simvastatin 20 mg once daily, atorvastatin 20 mg once daily, or rosuvastatin 20 mg once daily. The duration of statin treatment was up to 5 days in the period of preoperation. Using a retrospective cohort design, we identified 136 patients who underwent cardiac valve replacement at Sun Yat-sen Memorial Hospital between June 1, 2014, and January 1, 2016, with a preoperative diagnosis of RHD. The ethics committee of Sun Yat-sen Memorial Hospital approved the study. The primary outcome variables were perioperative death, reoperation, and pleural puncture. The secondary outcome variables were the CPB time, aortic cross-clamp time, drainage time, ICU stay, and hospital stay. The perioperative period included the preoperative period, intraoperative period, anesthesia recovery period, and postoperative period until either discharge or death in patients who were enrolled in this study. All of the patients agreed to participate in this study and the median follow-up time was 1 month.

### 2.2. Selection of Patients and Assessments

Clinical records and the perioperative period of survival of patients with RHD were reviewed. Diagnosis of RHD was based on the Jones criteria and, with some modifications and revisions, established by American Heart Association (AHA). Here, echocardiographic changes for structural and functional abnormality of the heart valves that meet the following criteria, with a history of definite acute rheumatic fever (ARF) or being considered to be rheumatic in origin excluding other etiologies, are considered to represent RHD [10, 11]. The criteria for guidance in the diagnosis of RHD, based on available clinicians' notes, symptoms, clinical history, and echocardiogram reports, are as follows: (1) pathological mitral regurgitation or aortic regurgitation, plus at least two morphological features, including the anterior mitral valve leaflet thickening, restricted leaflet motion, and excessive leaflet tip motion during systole, conducted by mitral valve; (2) mitral stenosis mean gradient ≥ 4 mmHg; (3) pathological aortic regurgitation and at least two morphological features, including the bicuspid aortic valve and aortic root dilatation [10–12]. In addition, exclusion criteria were as follows: (1) estimated glomerular filtration rate (eGFR) < 30 ml/min according to the Cockcroft-Gault formula; (2) age < 18 years; (3) no RHD in the clinical diagnosis; (4) no valve replacement surgery; and (5) no CPB. Eligible patients were classified as having statin therapy and nonstatin therapy in the normal or impairment renal function subgroups. In our study, the diagnosis of RHD was determined according to the 2012 World Heart Federation criteria [12].

**Figure 1:**
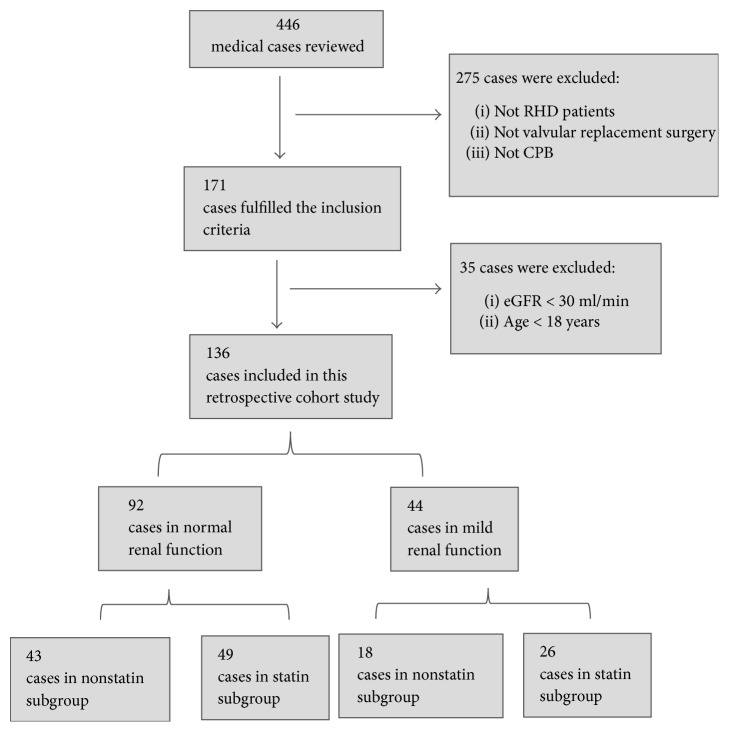
The analysis strategy of this retrospective cohort study.

The Cockcroft-Gault equation was used to calculate the eGFR (ml/min/1.73 m^2^) [13]. This equation was calculated as follows: eGFR = [(140 − age) × (weight in kg) × (0.85 if female)]/[72 × serum creatinine (SCr)]. According to the Cockcroft-Gault equation, we classified the patients into 2 groups: (1) normal renal function, eGFR of ≥80 ml/min/1.73 m^2^, and (2) renal function impairment, eGFR less than 80 ml/min/1.73 m^2^. In our unit, chest or pericardial tubes were removed under the following conditions: (1) drainage output declined to less than 250 mL in a 24-hour period; (2) there were no clinical signs and symptoms of postpericardiotomy syndrome; and (3) patients were in a good general condition. Additionally, ultrasound, X-rays, and computed tomography scans were used to assess pleural fluid volume and characteristics, according to the surgical procedure of the World Health Organization criteria [14–16]. The analysis strategy is shown in Figure 1.

### 2.3. Statistical Analysis

Baseline characteristics of the patients were classified by categories of renal function. We recorded age, sex, body mass index (BMI), current smoking status, left ventricular ejection fraction (LVEF), erythrocyte sedimentation rate (ESR), antistreptolysin O, diabetes mellitus, hypertension, New York Heart Association (NYHA) functional class III or IV, and levels of brain natriuretic peptide (BNP), hemoglobin (Hb), platelets (PLT), C-reactive protein (CRP), glutamate oxaloacetate transaminase (AST), and alanine aminotransferase (ALT). Continuous variables are presented as mean (SD) and minimum and maximum values. Between-group comparisons were tested by ANOVA. For comparison of the ESR, PLT, Hb, and plasma concentrations of CRP, AST, and ALT between the different subgroups, the Wilcoxon rank sum test was performed. Categorical variables are shown as counts and percentages, with between-group comparison by the *χ*^2^ or Fisher's exact test. For missing data, we used the mean imputation method to avoid statistical bias owing to data that were missing completely at random and the number of data was greater than 10. For patients in whom survival could not be assessed, we used the deletion method to remove it in statistical process.

Because this was a retrospective cohort study with subgroup analysis, the odds ratios (ORs) or mean differences and 95% confidence intervals (CIs) were viewed cautiously using the exact Clopper and Pearson confidence interval method. A 2-sided 5% statistical significance level was used.

## 3. Results

### 3.1. Patients' Baseline Characteristics

A total of 136 patients with RHD who underwent cardiac surgery were included in our study according to the inclusion criteria. We included 92 patients with normal renal function [creatinine clearance (CrCl): ≥80 ml/min] and 44 patients with renal impairment (CrCl: 30–79 ml/min). Among patients with normal renal function, 43 and 49 received statin and nonstatin treatment, respectively. Among patients with renal impairment, 18 and 26 received statin and nonstatin treatment, respectively. There were 3 deaths, 2 patients underwent reoperation, and 25 patients underwent thoracentesis in the course of the study period.

There were significant differences in gender between the renal function subgroups (Fisher's exact test, *P* < 0.01) and Hb levels (Wilcoxon rank sum test, *P* = 0.01). Among patients with RHD in our cohort, 8.8% had diabetes at baseline, 27.2% had hypertension at baseline, and 38.4% had NYHA functional class III/IV at baseline. The other baseline factors (Age, BMI, LVEF, ESR, BNP, CRP, PLT, ALT, AST, and previous medications) are shown in Table 1.

### 3.2. Perioperative Outcomes according to Baseline Renal Function

There were significant differences in the drainage time (difference in means: 1.0; 95% CI: 0.88, 1.12; *P* < 0.01), ICU stay (difference in means: 0.2; 95% CI: 0.17, 0.23; *P* = 0.02), and hospital stay (difference in means: 6.6; 95% CI: 6.15, 7.05; *P* < 0.01) between the normal renal function group and the renal impairment group. There were no significant differences in CPB time (difference in means: 5.4; 95% CI: 3.17, 7.63; *P* = 0.76) and aortic cross-clamp time (difference in means: −0.6; 95% CI: −2.38, 1.18; *P* = 0.51) between the normal renal function group and the renal impairment group.

Among patients in the normal renal function group, 2.2% (2/92) underwent reoperation. In this group, there were 2 (2.2%) deaths at postoperation. There were no significant differences in these variables between the two subgroups (reoperation, OR: 0.40; 95% CI: 0.02, 8.46; *P* = 0.56; perioperative mortality, OR: 1.02; 95% CI: 0.09, 11.59; *P* = 0.97). 14 (31.8%) patients underwent thoracic puncture in the postoperative period in the renal impairment group, and this number was significantly higher than that in the normal renal function group (OR: 3.33; 95% CI: 1.36, 8.11; *P* < 0.01) (Table 2).

### 3.3. Perioperative Outcomes of Statin and Nonstatin Treatments Related to Renal Function

In the normal renal function group, there were 43 patients in the nonstatin subgroup and 49 in the statin subgroup. There were no significant differences in the drainage time (difference in means: −0.30; 95% CI: 0.38, −0.22; *P* = 0.37), ICU stay (difference in means: −0.20; 95% CI: −0.24, −0.16; *P* = 0.13), hospital stay (difference in means: −1.80; 95% CI: −2.05, −1.55; *P* = 0.15), and thoracic puncture at postoperation (OR: 1.06; 95% CI: 0.3, 3.76; *P* = 0.59) between the subgroups.

**Figure 2:**
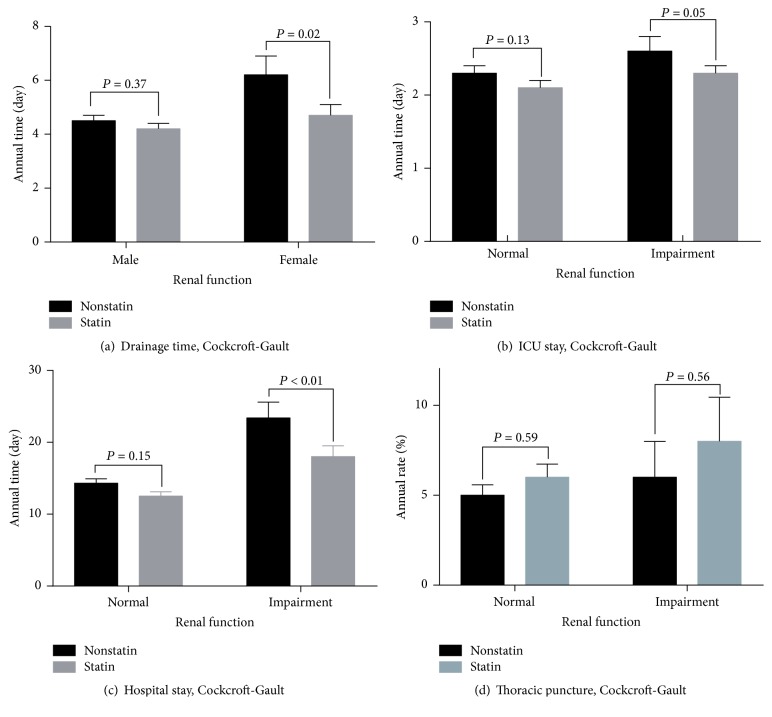
Perioperative outcomes in statin therapy related to renal function. ICU indicates intensive care unit; eGFR, estimated glomerular filtration rate.

In the renal impairment group, there were 18 patients in the nonstatin subgroup and 26 in the statin subgroup. There were significant differences in the drainage time (difference in means: −1.50; 95% CI: −1.86, −1.14; *P* = 0.02), ICU stay (difference in means: −0.30; 95% CI: −0.40, −0.20; *P* = 0.05), and hospital stay (difference in means: −5.40; 95% CI: −6.57, −4.23; *P* < 0.01) in patients who received nonstatin treatment compared with patients who received statin treatment. However, there was no significant difference in thoracic puncture (OR: 0.89; 95% CI: 0.25, 3.22; *P* = 0.56) between the subgroups. The analysis results are shown in Figure 2.

## 4. Discussion

RHD can cause cumulative damage to heart valves during episodes of acute rheumatic fever because of an inadequate response to invasive group A streptococcal infection. The cornerstone of pathophysiology has long been described as “trilogy,” including group A streptococcal strain infection, aberrant host immune response, and cross-reaction with host tissue proteins [10, 12]. In this seminar, these cross-reactions gave rise by both antibody and T-cell responses triggering an immune-inflammatory response leading to permanent and irreversible heart valve damage in genetically susceptible host. What is more, pathological mitral valve incompetence (including the mitral stenosis or mitral regurgitation) is the commonest valvular lesion, occurring in a higher risk in rheumatic damage than aortic valve and tricuspid valve [11]. Furthermore, valvular damage can cause hemodynamic changes, thus leading the ventricular hypertrophy, atrial fibrillation (AF), stroke, and systemic embolization. Some large, randomized, controlled trials have suggested that CKD is independently associated with coronary heart disease, heart failure, peripheral artery disease, venous thromboembolism, hypertension, left ventricular hypertrophy, AF, and cardiovascular disease, and it increases the risk of stroke, thromboembolism, and major bleeding [17].

CKD involves damage to the kidney with a reduction in eGFR < 60 ml/min/1.73 m^2^ for 3 months or longer, and the morbidity is estimated as 8%–16% worldwide [18]. Increasing evidence has shown that CKD is associated with an increasing incidence of cardiovascular events because of uremic toxins [19]. Furthermore, uremic toxins have emerged as a major factor for explaining cardiovascular disease. Notably, use of CPB is associated with an increase in the risk of glomerular and tubular injury because of the systemic inflammatory response and renin–angiotensin–aldosterone system activation, especially leading to impairment of kidney function. Therefore, disease of the heart or kidney often involves dysfunction and injury to other organs. Notably, we found that renal impairment was associated with a longer time of drainage, ICU stay, and hospital stay and a higher risk of thoracic puncture in the perioperative period compared with normal renal function in patients with RHD who underwent cardiac surgery.

Previous studies have suggested that statins are HMG-CoA reductase inhibitors and they increase the expression of vascular endothelial cell surface low-density lipoprotein (LDL) receptors, leading to increased uptake of LDL from the circulation. Statins have therapeutic effects because of anti-inflammatory and antioxidant properties, and they stimulate and upregulate endothelial nitric oxide synthase. In our retrospective cohort study that compared statin and nonstatin treatments, in the early postoperative period, there was a large decline in the time of drainage, ICU stay, and hospital stay, as well as a risk of thoracic puncture in the statin treatment group patients with renal impairment.

There are no standard criteria for the timing of drain removal. Most previous studies on this issue have suggested that removing the drainage tubes as soon as possible is safe if there are no related signs of active bleeding and pericardial incision syndrome. There is a 2-fold effect between drainage and pericardial effusion at postoperation [20]. When drainage removal is too early, persistent effusion may lead to accumulation of blood or inflammatory fluids in the pericardium and mediastinum. This can cause pleural puncture and reoperation at postoperation. However, early removal of tubes can reduce irritation and facilitate the patients' recovery [20]. Therefore, determining the appropriate time to remove the tubes is difficult. In cardiac surgery, the optimal timing of when drainage removal should be performed following the operation is a decline in drainage output to less than 50 ml in a 5-hour period or less than 80 ml in an 8-hour period [20, 21]. However, most patients with valvular disease are complicated by arrhythmia, especially in RHD. Many of these patients may take aspirin, clopidogrel, or warfarin to prevent blood clots at preoperation. Additionally, the history of medication may be an important factor in prolonging the time of drainage, especially in patients with AF who have routine application of low molecular weight heparin. In our study, most of the patients had a history of anticoagulant or antithrombosis therapy (48.5% patients took aspirin, 15.4% took clopidogrel, 9.6% took warfarin or other drugs, and patients with AF had injection of low molecular weight heparin). In several studies, drainage time was associated with a prolonged ICU stay and hospital stay because of complications of systemic inflammation, respiratory dysfunction, and infection [22, 23]. Of the drainage removal, our study criteria are in accordance with those of Smulders et al. [20, 21].

In cardiac surgery, statins are widely recommended for reducing LDL concentrations in the circulation and for decreasing the inflammatory response to maintain normal physiological function. Our study aimed to evaluate the efficacy of statin treatment in the perioperative period related to baseline renal function in patients with RHD undergoing cardiac surgery. As mentioned above, there were significant differences in the drainage time, ICU stay, hospital stay, and the risk of thoracic puncture between the renal function groups. However, our study was not adequately powered to statistically determine any differences in CPB time, aortic cross-clamp time, rate of reoperation, and rate of perioperative mortality between the renal function groups. Additionally, in the statin therapy subgroup analysis, we found that statin treatment was associated with a shorter time of drainage, ICU stay, and hospital stay than nonstatin treatment in patients with renal impairment, but not in those with normal renal function.

## 5. Study Limitations

Our study has several limitations. First, the present study was limited because this was single-center, retrospective analysis that was conducted in a small population of Chinese patients. Therefore, multicenter, randomized, controlled studies with a longer duration follow-up are required in a larger population to completely understand the efficacy of statin treatment related to baseline renal function in patients with RHD undergoing cardiac surgery. Second, in the present study, we excluded patients with an eGFR < 30 ml/min and age < 18 years. In fact, in our unit, we experienced a small number of patients with RHD and an eGFR < 30 ml/min, but these patients could not be assessed for further analysis. Additionally, apart from other notable factors, age is an established important factor for postoperative recovery in patients with cardiac surgery. Third, there were many factors, such as operation mode, size, and positioning of the tubes, and tube patency, which determined the efficiency of drainage. In fact, there was a relatively fixed pattern in diagnosis, surgery, and management of medication in our unit. Understanding the interaction of such factors is important for accurately assessing the drainage time.

## 6. Conclusion

Our retrospective cohort study suggests that patients with renal impairment have an increased risk of thoracic puncture and a prolonged time of drainage, ICU stay, and hospital stay. For patients with renal impairment, there is a significant reduction in drainage time, ICU stay, and hospital stay for statin therapy in patients with RHD who have cardiac surgery. These results may provide a reference for perioperative management of Chinese patients undergoing cardiac surgery.

## Figures and Tables

**Table 1:** Baseline characteristic of RHD patients with significant associations with eGFR.

Characteristic	Overall (*n* = 136)	CrCl ≥ 80 ml/min (*n* = 92)	CrCl 50–79 ml/min (*n* = 44)	*P* value
Age, y				
Mean (SD)	56.2 (13.5)	57.7 (13.4)	53.0 (13.2)	0.89
Minimum, maximum	19, 91	19, 91	22, 75	
Female sex, %	59.6	46.7	86.4	0.01
BMI				0.03
Mean (SD)	22.8 (4.4)	23.1 (4.7)	22.0 (3.7)	
Minimum, maximum	14, 39	14.0, 39.0	16.0, 34.0	
LVEF				
Mean (SD)	60.4 (10.1)	59.7 (10.9)	62.0 (8.0)	0.76
Minimum, maximum	29.0, 75.0	29.0, 75.0	45.0, 75.0	
ESR				0.77
Mean (SD)	17.8 (12.5)	16.1 (11.7)	18.6 (16.4)	
Minimum, maximum	3, 77	2.0, 60.0	3.0, 77.0	
Antistreptolysin “O”				0.95
Mean (SD)	83.6 (70.2)	78.7 (73.0)	94.8 (63.5)	
Minimum, maximum	11, 365	11, 365	17, 261	
BNP				
Mean (SD)	1808.8 (2213.6)	1873.1 (2300.6)	1674.2 (2038.3)	0.63
Minimum, maximum	7.2, 9236.0	7.2, 9036.0	21.7, 6778.0	
Hb				
Mean (SD)	132.5 (22.1)	136.8 (21.7)	123.5 (20.4)	0.01
Minimum, maximum	65.0, 259.0	69.0, 259.0	65.0, 155.0	
PLT				
Mean (SD)	201.8 (62.2)	203.4 (60.8)	198.4 (65.7)	0.66
Minimum, maximum	70.0, 495.0	70.0, 495.0	116.0, 444.0	
CRP				
Mean (SD)	10.7 (13.4)	10.7 (13.2)	10.7 (14.1)	0.99
Minimum, maximum	2, 76	2.0, 71.0	2, 76	
ALT				
Mean (SD)	26.5 (21.8)	26.6 (19.1)	26.3 (26.7)	0.94
Minimum, maximum	8.0, 173.0	8.0, 173.0	9.0, 66.0	
AST				
Mean (SD)	28.1 (23.6)	28.9 (26.5)	26.1 (15.8)	0.52
Minimum, maximum	6.0, 189.0	6.0, 189.0	8.0, 69.0	
NYHA class III/IV, %	38.40%	37.50%	39.20%	0.67
Hypertension, %	27.2	26.1	29.5	0.69
Diabetes mellitus, %	8.8	8.7	9.1	0.47
Medications				
ASA, %	47.8	44.8	48.8	0.32
ARB, %	33.1	33.3	32.1	0.54
ACEI, %	28.1	29.2	27.5	0.44
ß-blocker, %	46.7	47.7	44.3	0.17
Warfarin, %	4.7	4.5	4.9	0.74
Clopidogrel, %	13.9	14.6	13.2	0.58

BMI indicates body mass index; ESR, erythrocyte sedimentation rate; LVEF, left ventricular ejection; BNP, brain natriuretic peptide; PLT, platelet; CRP, C reactive protein; ALT, alanine aminotransferase; AST, glutamate oxaloacetate transaminase; Hb, hemoglobin; NYHA class, New York Heart Association functional class; ARB, angiotensin receptor blocker; ACEI, angiotensin-converting enzyme inhibitor.

**Table 2:** Perioperative outcomes according to baseline renal function.

Items	CrCl ≥ 80 ml/min (*n* = 92)	CrCl 50–79 ml/min (*n* = 44)	DM or OR (95% CI)	*P* value
CPB-time, min	142.2 ± 5.2	147.6 ± 6.7	−0.94 (−1.31, −0.56)	0.76
Aortic cross-damp time, min	96.7 ± 3.8	96.1 ± 5.5	0.14 (−0.22, 0.49)	0.81
Drainage time, day	4.3 ± 0.2	5.3 ± 0.4	−3.55 (−4.11, −2.99)	<0.01
ICU stay, day	2.2 ± 0.1	2.4 ± 0.1	−3.03 (−3.55, −2.52)	0.02
Hospital stay, day	13.2 ± 0.4	19.8 ± 1.5	−7.20 (−8.14, −6.26)	<0.01
Reoperation (*n*, %)	2 (2.2%)	0.0 (0)	2.46 (0.12, 52.3)	0.56
Thoracentesis (*n*, %)	11 (12.0%)	14 (31.8%)	0.29 (0.12, 0.71)	<0.01
Perioperative mortality (*n*, %)	2 (2.2%)	1 (2.3%)	0.96 (0.08, 10.83)	0.97

CPB indicates cardiopulmonary bypass; ICU, intensive care unit; 95% CI, 95% confidence interval; DM, Difference in Means; OR, Odds Ratio.

## Supplementary Materials

Supplementary Materials STROBE Statement—checklist of items that should be included in reports of observational studies.
