# Supplementary material for: Efficacy of Statin Therapy Related to Baseline Renal Function in Patients with Rheumatic Heart Disease Undergoing Cardiac Surgery
Source: Biomed Res Int. 2018 Apr 15;2018:5972064. doi: 10.1155/2018/5972064 (PMC5925190; doi:10.1155/2018/5972064)
Supplement: Supplementary Materials — STROBE Statement—checklist of items that should be included in reports of observational studies. [file 5972064.f1.doc]

STROBE Statement—checklist of items that should be included in reports of observational studies

| **Item** | No | Recommendation |
| --- | --- | --- |
| **Title and abstract** | 1 | (a) We performed a retrospective study to determine the perioperative benefit of statin treatment related to baseline renal function in rheumatic heart disease(RHD) patients with cardiac surgery |
|  |  | (*b*) For the renal function patients, there was the higher risk of thoracic puncture, and longer time of drainag, ICU stay, hospital stay in comparison of normal renal function. Furthermore, statins was associated with reduction of drainage time, ICU stay, and hospital stay in patients with renal function, but not in normal renal function. |
| Introduction | | |
| Background/rationale | 2 | The early stages of CKD were significantly associated with higher risk of cardiovascular disease due to fluid overload, electrolyte abnormalities, and local micro-inflammation. Statins has been expected to reduce the risk of acute renal injury following cardiac surgery, as well as clinical guidelines recommend, which play an important role with its antioxidant and anti-inflammatory effects. While, the clinical benefits in the perioperation of cardiac surgery with renal function impairments were not clear. |
| Objectives | 3 | Our aim was to determine the perioperative benefit of statin treatment related to baseline renal function in RHD patients with cardiac surgery. |
| Methods | | |
| Study design | 4 | The present study was a single-center retrospective cohort study. |
| Setting | 5 | Statins treatment duration was up to 5 days in the postoperation. Utilizing a retrospective cohort design, we identiﬁed 136 patients who underwent cardiac valvular replacement at Sun-Yat Sen Memorial Hospital between June 1, 2014, and January 1, 2017 with a preoperative diagnosis of RHD. |
| Participants | 6 | (*a*) *Cohort study*—The exclusion criteria were as follows:(1) Estimated glomerular filtration rate (eGFR) <50 ml/min according to Cockcroft-Gault; (2) Age < 18 years; (3)Not RHD in the clinical diagnosis; (4) Not valvular replacement surgery; (5) Not CPB. |
|  |  | (*b*) *Cohort study*—Patients in statin group were administered simvastatin 20mg once daily, atorvastatin 20mg once daily, or rosuvastatin 20mg once daily. Statins treatment duration was up to 5 days in the postoperation. Furthermore, Cockcroft–Gault equation was used to calculate the eGFR. |
| Variables | 7 | In our study, the diagnosis of RHD were as follow the2012 World Heart Federation criteria. The primary outcome variable were the perioperative death, reoperation, pleural puncture. The second outcome variable were the CPB-time and aortic cross-damp time, drainage time, ICU stay, hospital stay. The perioperative period were included the preoperative period, intraoperative period, anesthesia recovery period, and postoperative period until either discharge or death in patients enrolled in this study. |
| Data sources/ measurement | 8* | Clinical records and perioperative period survival of RHD patients were reviewed. According to the Cockcroft–Gault equation, we classified the patients into the two groups, (1) normal renal function: eGFR of ≥ 80 ml/min/1.73 m^2^; (2) renal impairment: eGFR if 50 to 80 ml/min/1.73 m^2^. Chest or pericardial tubes were removed as follows: (1) the drainage output declined to less than 250 mL in a 24-hour period; (2) No clinical signs and symptoms of postpericardiotomy syndrome; (3) Patients in a good general condition. Additionally, ultrasound, X-rays and CT scans has been used to assess pleural fluid volume and character, guiding the surgical procedure utilized World Health Organization (WHO) criteria. |
| Bias | 9 | There are two author collected clinical data and three different authors performed the statistical analysis. Additionally, Sun Yat-Sen University medical statistics provide the statistical consultation and support to avoid potential sources of bias. |
| Study size | 10 | In this study, we identiﬁed 136 patients who underwent cardiac valvular replacement in our unit with a preoperative diagnosis of RHD. |
| Quantitative variables | 11 | Continuous variables were presented as means (SD), minimum and maximum, with between-group comparisons tested by ANOVA. For comparison of ESR, PLT, Hb, CRP, AST, and ALT plasma concentrations between the different subgroups, the Wilcoxon rank sum test was performed. Categorical variables were presented as counts and percentages, with between-group compared by ᵪ^2^ or Fisher’s exact tests. |
| Statistical methods | 12 | (*a*) ANOVA, Wilcoxon rank sum test, ᵪ^2^ and Fisher’s exact tests. |
|  |  | (*b*) Wilcoxon rank sum test, ᵪ^2^ and Fisher’s exact tests. |
|  |  | (*c*) Used deletion method owing to the missing completely at random and the number was greater than 10. For the patients can not been assess the survival conditions, we used the deletion method to remove it. |
|  |  | (*d*) *Cohort study*—Of the missing data, some data were irregular inspection, and some patient's telephone number were changed. We were used mean imputation and deletion method to address it. |
|  |  |  |

Continued on next page

| Results | | |
| --- | --- | --- |
| Participants | 13* | (a) A total of 136 RHD patients underwent cardiac surgical cases were included in our study according to inclusion criteria; 92 patients with normal renal function(CrCl≥80ml/min), and 44 patients with renal function(CrCl 50-79 ml/min). Of the normal renal function patients, 43 and 49 patients were received the statin and non-statin treatment, respectively. |
|  |  | (b) There were 110 patients not include following the exclusion criteria. |
|  |  | (c) The flow diagram is presented in Figure 1. |
| Descriptive data | 14* | (a) Among RHD patients in our cohorts, 8.8% had diabetes baseline, 27.2% had hypertension baseline, 38.4% had New York Heart Association (NYHA) functional class III/IV baseline. The other baseline factors (Age, BMI, LVEF, ESR, BNP, CRP, PLT, ALT, AST and previous medications) are presented in Table 1. |
|  |  | (b) There was 1 patient removed for missing the contact information, and 12 patients missing the some irregular inspection. |
|  |  | (c) *Cohort study*—the median follow-up time was1 month. |
| Outcome data | 15* | *Cohort study*—There were 3 deaths, 2 patients underwent reoperation, and 25 patients underwent thoracentesisin the course of the study period. Furthermore, the drainage time, ICU stay, hospital stay, CPB-time, and Aortic cross-damp time et al. are presented in Table2 and Figure 2. ( Related to baseline renal function) |
| Main results | 16 | (*a*) For the renal function patients, there was the higher risk of thoracic puncture [odds ratio(OR), 0. 29; 95% confidence interval(CI), (0.12,0.7), P<.01] and longer time of drainage [Differences in means,-3.55; 95%CI,(-4.11,-2.99),P<.01], ICU stay [Differences in means, -3.03;95%CI, (-3.55,-2.52), P=.02], hospital stay [Differences in means,-7.20; 95%CI, (-8.14,-6.26), P<.01] in comparison of normal renal function. Furthermore, statins was associated with reduction of drainage time[Differences in means, 1.40; 95%CI, (0.20,2.59), P=.02], ICU stay [Differences in means, 0.30; 95%CI, (0.01,0.61), P=.05], and hospital stay [Differences in means,5.44; 95%CI, (1.98,8.91), P<.01]in patients with renal function(interaction P≤.05 for all), but not in normal renal function. |
| Other analyses | 17 | There was notable difference in the drainage time (Difference in Means 1.40, 95%CI 0.20, 2.59, P=.02), ICU stay (Difference in Means 0.30, 95%CI 0.01, 0.61, P=.05), hospital stay (Difference in Means 5.44, 95%CI 1.98, 8.91, P<.01) in patients who received Non-statin treatment compared with patients who received Statin treatment. However, there was no significant difference in thoracic puncture (OR 1.13, 95%CI 0.31,4.07, P=.56) between groups. |
| Discussion | | |
| Key results | 18 | The patients with renal function increase the risk of thoracic puncture and prolonged time of drainage, ICU stay, hospital stay .For the renal function patients, a significant reduction in drainage time, ICU stay, and hospital stay for statins therapy in RHD patients with cardiac surgery. |
| Limitations | 19 | First, the present study was limited because this a single-unit retrospective analysis conducted in a small population of Chinese patients. Second, in the present study, we excluded the patients with eGFR<50ml/min and age <18 years. Third, there were many factors, such as operation mode, size and positioning of the tubes, tube patency, determine the efficiency of drainage. |
| Interpretation | 20 | For the limitation, actually, there was a relatively fixed pattern in diagnosis, surgery, and medication management in our unit. We collected a small number of RHD patients with eGFR<50ml/min, and cannot be assessed such patients for the further analysis. In addition, apart from other notable factors, age is an established important factor for postoperative recovery in patients with cardiac surgery. |
| Generalisability | 21 | These results may provide the reference message for the perioperative management of Chinese patients undergoing cardiac surgery. |
| Other information | | |
| Funding | 22 | This work was supported by the National Natural Science Foundation of China (NSFC No.81771165), the Natural Science Foundation Project in Guangdong province, China (Grant No. 2016A030313295), the Major Project Development and Emerging, Interdisciplinary Funding Projects of Sun Yat-sen University (Grant No. 15ykjc17b) and Guangzhou science and technology project of Major Special Research Topics on International Collaborative Innovation (Grant No. 201704030032). |
